# Supplementary material for: Monitoring the Disulfide Bonds of Folding Isomers of Synthetic CTX A3 Polypeptide Using MS-Based Technology
Source: Toxins (Basel). 2019 Jan 17;11(1):52. doi: 10.3390/toxins11010052 (PMC6356385; doi:10.3390/toxins11010052)
Supplement: Supplementary file 1 [file toxins-11-00052-s001.pdf]

Supplementary Materials: Monitoring the Disulfide Bonds of Folding Isomers of Synthetic CTX A3 Polypeptide Using MS-Based Technology

Sheng-Yu Huang, Tin-Yu Wei, Bin-Shin Liu, Min-Han Lin, Sheng-Kuo Chiang, Sung-Fang Chen and Wang-Chou Sung

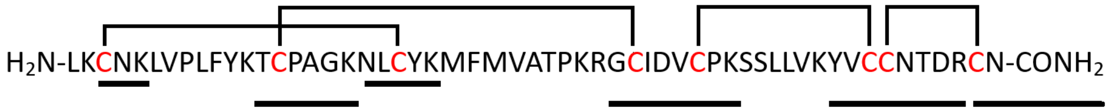

Figure 1. Amino acid sequence of synthetic polypeptide with an amidated C-terminal asparagine residue. The solid line linkage between cysteine residues represents the native disulfide bonds found in the native CTX A3 toxin. The bottom line indicates the tryptic fragment linked by these disulfide bonds

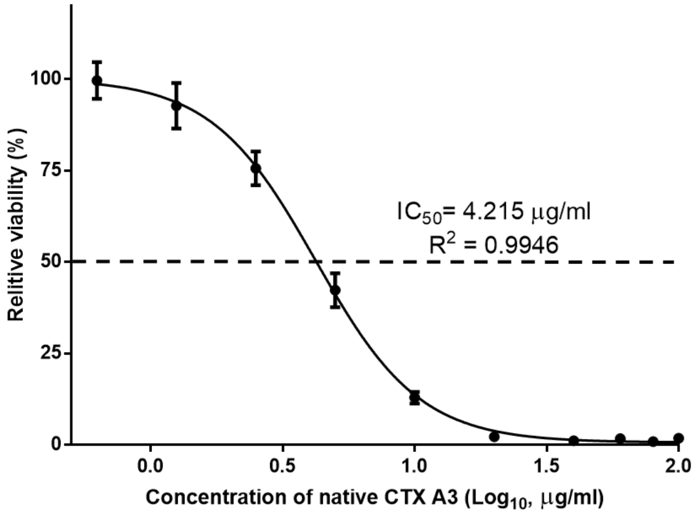

Figure S2. The cytotoxic activity of native CTX A3 toxin on the HL-60 cells. Results were acquired from experiments performed in triplicate. The dashed line indicates the toxin concentration that caused death of half of the cells.
